# Modulation of Phospho-CREB by Systemically Administered Recombinant BDNF in the Hippocampus of the R6/2 Mouse Model of Huntington's Disease

**DOI:** 10.1155/2019/8363274

**Published:** 2019-02-06

**Authors:** Emanuela Paldino, Carmela Giampà, Elena Montagna, Cecilia Angeloni, Francesca R. Fusco

**Affiliations:** ^1^IRCCS Fondazione Santa Lucia, Laboratory of Neuroanatomy, Rome, Italy; ^2^Catholic University of Rome Department of Anatomy, Rome, Italy

## Abstract

Huntington's disease (HD) is an autosomal dominant neurodegenerative disease due to an expansion of a trinucleotide repeats in* IT15* gene encoding for the protein huntingtin. Motor dysfunction, cognitive decline, and psychiatric disorder are typical clinical signs of HD. In HD, mutated huntingtin causes a major loss of brain derived neurotrophic factor (BDNF), causing striatal atrophy. Moreover, a key involvement of BDNF was observed in the synaptic plasticity that controls the acquisition and/or consolidation of certain forms of memory. We studied changes in hippocampal BDNF and in CREB in the R6/2 mouse model of HD. Moreover, we investigated if the beneficial effects of systemically administered recombinant BDNF observed in the striatum and cortex had an effect also on the hippocampus. Osmotic minipumps that chronically released recombinant BDNF or saline solution from 4 weeks of age until euthanasia were implanted into R6/2 and wild type mice. Our data show that BDNF is severely decreased in the hippocampus of R6/2 mice, while BDNF treatment restored its physiological levels. Moreover, the chronic administration of recombinant BDNF promoted the increment of phosphorylated CREB protein. Our study demonstrates the involvement of hippocampus in the pathology of R6/2 model of HD and correlates the beneficial effects of BDNF administration with increased hippocampal levels of BDNF and pCREB.

## 1. Introduction

Huntington's disease (HD) is a neurodegenerative disorder characterized by motor dysfunction, cognitive decline, and emotional and psychiatric disorders [1–4]. Motor symptoms are dominated by chorea, an involuntary muscle contraction that results from the impairment of the basal ganglia, which is the main target of HD. These involuntary movements are nonstereotyped and irregular. The study of molecular mechanisms involved in the disease could represent an important opportunity to design new therapeutic strategies to treat or prevent motor symptoms and to manage psychological complications. The striatal part of the basal ganglia degenerates in HD. In particular, spiny projection neurons, which constitute about 95% of the striatum, degenerate massively in HD. However, signs of neurodegeneration are observed also in the cortex, thalamus, globus pallidus, amygdala, brainstem, and cerebellum. The extent of this cellular damage could explain the heterogeneity of HD clinical features [5]. Cortical pathology is also evident and contributes to the overall dramatic loss of brain volume (up to 40%) in the late stages of the disease. Moreover, signs of cortical dysfunction are often observed before neuropathological signs are apparent [6]. Another brain region that is involved in HD is the hippocampus. Indeed, hippocampus is a key structure of the limbic system and has been considered a mediator of learning and memory [7]. It has been described that impaired hippocampal neuronal plasticity gives rise to a severely depressed mood [8, 9]. Depressive disorders, as well as cognitive symptoms, characterize the presymptomatic stage of HD disease before the onset of motor changes [10, 11]. Moreover, impaired learning that occurs before motor symptoms has been described in several mouse models of HD [12–14]. These behavioral abnormalities are accompanied by deficits in hippocampal LTP [15–17]. Moreover, Gil et al. showed through elegant experiments a dramatic hippocampal cells loss due to an impairment of neurogenesis process in the mouse model of HD, R6/2 [18]. In HD, a consistent cell loss in the hippocampus was described in the CA1 subfield [19] and a decreased cell proliferation was also observed in the dentate gyrus [20]. Three-week-old mice carrying the HD mutation (Bates' R6/2 mice) develop neuronal nuclear inclusions of mutant huntingtin in the hippocampal CA1 region and progressively expand to DG and CA3 by 10 weeks [21]. Interestingly, long-term spatial and recognition memory deficits were described in a knock-in model of HD and associated with reduced hippocampal CBP levels and selective disruption of memory-related CREB/CBP-dependent genes [22]. Brain derived neurotrophic factor (BDNF) is a neurotrophin that is greatly affected in HD [23]. Aside from the prosurvival role for the striatum, which accounts for its great relevance in HD, BDNF promotes activity-driven actin polymerization in dendritic spines [24] and facilitates LTP induction by theta burst stimulation [25, 26]. Alterations of BDNF signaling pathway may involve modification of the spines cytoskeleton, which could result in the disruption of stable synaptic changes needed to encode memory. Interestingly, upregulated BDNF has shown to rescue synaptic plasticity in HD mice [27]. Furthermore, Kuipers and colleagues demonstrated the proneurogenic action of BDNF in the hippocampal neurogenesis, showing how BDNF-mediated signals are involved in the proliferation and integration of newborn cells in the adult hippocampal circuitry [28]. In this study, we investigated the changes in the hippocampus of the R6/2 mouse model of HD, with particular focus on CREB and BDNF, and how they are modulated by the administration of systemically delivered recombinant BDNF [29, 30]. The chronic administration of recombinant BDNF promoted the increment of CREB protein phosphorylation (pCREB) in the hippocampal regions where pCREB expression is severely impaired. Simultaneously the restored-BDNF expression ensured the neuroprotection by improving the performances of R6/2 compared to untreated mice.

## 2. Experimental Procedures

### 2.1. Animals and Surgery

All animal experiments which satisfy ARRIVE guidelines were performed in accordance with European Communities Council Directive (2010/63 EU) as adopted by the Santa Lucia Foundation Animal Care and Use and approved by Italian Ministry of Health. Heterozygous transgenic R6/2 males of CBAXC57BL/6 strain were and bred with CBAXC57BL/6 F1 females, all obtained from Jackson Laboratories (Bar Harbor, ME). We used F1 mice for all experiments, to minimize variations in CAG number and in phenotype. In this study, males and females were employed, and no differences were found between genders. After being weaned and genotyped, mice underwent treatment at 4 weeks of age. We used the following groups: R6/2 mice treated with saline, R6/2 mice treated with BDNF, and wild type mice treated with saline. Recombinant BDNF was not administrated in wild type for this study mice, as previous studies did not show a statistically significant modulation of endogenous BDNF after chronic treatment [29, 30]. Recombinant BDNF (Regeneron Pharmaceuticals) was diluted in PBS with 0.1% bovine serum albumin (protease-free, Sigma Aldrich, St Louis, MO) to a dosage of 4.0 *μ*g per 24 h (152 *μ*g in100 *μ*l per micropump) with 0.1% bovine serum albumin (protease- free, Sigma Aldrich, St Louis, MO). Anesthesia was performed by xylazine and zolazepam + tyletamine before surgery. Chronic, indwelling osmotic minipump (Alzet Model 1004; Durect, Cupertino, CA) containing either recombinant BDNF or saline was implanted subcutaneously. Following the previously described procedure [29, 30]. Experiments were performed under the same conditions by one investigator at the same time of the day. Mice were identified by a randomly assigned code, housed five in each cage under standard conditions with ad libitum access to food and water. Investigators who were blinded collected all histological and molecular data to treatment.

### 2.2. Histological and Immunohistochemical Studies

#### 2.2.1. Tissue Processing

Twenty-seven animals (9 R6/2 treated with BDNF, 9 R6/2 treated with saline, and 9 wild type treated with saline) 13 weeks of age were transcardially perfused under deep anesthesia with saline solution containing 0.01 ml heparin. Half of the brains were postfixed in 4% paraformaldehyde and then cryoprotected in 20% and 30% sucrose, frozen in n-pentane (−45°C) for 3 minutes, and stored at −80°C until use. The other halves were dissected and the hippocampus, striatum, and cortex were isolated, frozen on dry ice, and stored at −80°C for molecular experiments. The whole hemispheres were dissected. For the histological and immunohistochemical experiments, brain sections between the bregma -2.30 and -2.56 were used in order to compare all cases. Sectioning was performed on a sliding frozen microtome at 40 *μ*m thickness. Primary omission controls, normal mouse, and rabbit serum controls and preimmune serum controls were used to confirm the specificity of the immunolabeling in our system.

#### 2.2.2. Analysis of CREB Activation in the Hippocampal Neurons

Double label immunofluorescence was employed to identify the intensity of activated CREB [31, 32] in the hippocampal neurons. Briefly, sections were incubated with a cocktail of NeuN antibody (mouse anti-NeuN, Immunological Science, Rome, Italy) at 1:500 concentration and the antibody against Ser-133 phosphorylated CREB (rabbit anti-Phospho-CREB, Upstate, NY) at 1:300 concentration in a 0.1 M phosphate buffered (PB) solution containing Triton X 0.3% and 0.02 sodium azide for 72 h at + 4°C. After 3 rinses in PB, 15 min each, sections were incubated with a cocktail of goat anti-rabbit Cy2-conjugated secondary antibody and donkey anti-mouse Cy3-conjugated secondary antibody (both Jackson Immunoresearch, West Grove, PA, USA) both were used at a concentration of 1:200, for 2 h at room temperature. Brain sections were mounted on gelatin-coated slides, cover slipped with GEL-MOUNT^TM^, and examined under an epi-illumination fluorescence microscope (Zeiss Axioskop 2), and a CLSM (Zeiss LSM 700). The acquisition setting was the same for all samples and images were acquired under no saturating exposure conditions. The gain and laser power were set to values that allowed an optimal visualization of the fluorophore used as secondary antibody and standardized using sections from wild type mice. The intensity of pCREB staining was calculated in each of three 1.0 mm square confocal microscope fields of mice from each saline, systemic BDNF treated R6/2 mice, and wild type littermates.

#### 2.2.3. Analysis of Brain Derived Neurotrophic Factor (BDNF) in the Hippocampal Neurons

Levels of BDNF in the hippocampal neurons after systemic BDNF administration were investigated by a double label immunofluorescence employing an antibody against BDNF (anti-rabbit BDNF, Immunological Sciences, Italy) at 1:500 concentration and NeuN antibody (mouse anti-NeuN, Immunological Science, Rome Italy) at 1:500 concentration in a 0.1 M phosphate buffered (PB) solution containing Triton X 0.3% and 0.02 sodium azide for 72 h at + 4°C. After 3 rinses in PB, sections were incubated with a cocktail of goat anti-mouse Cy2-conjugated secondary antibody and donkey anti-rabbit Cy3-conjugated secondary antibody (both Jackson Immunoresearch, West Grove, PA, USA) at a 1:200 concentration, for 2 h at room temperature. The images were acquired by CLSM ZEISS LSM700 laser scanning confocal microscope. The images were exported in TIF file format. BDNF immunoreactive hippocampal neurons were quantified by means of NIH Image J software. The intensity of fluorescent BDNF immunolabeling in NeuN positive hippocampal neurons was calculated in each of three 1.0 mm square confocal microscope fields of mice from each group

### 2.3. Neuropathological (Primary) Outcome Measures

#### 2.3.1. Evaluation of Hippocampal Neurons Area

Standard NeuroTrace green fluorescent Nissl stain was employed to evaluate the area of single hippocampal neuron in CA1, CA3, and DG, in each of three separate fields (one dorsolateral, one central, and one medial, each 1 mm in diameter) on each hemisphere in each of three rostrocaudally spaced sections from mice of each experimental group. Neurons were labeled with NeuroTrace (ThermoFisher Scientific, Rome, Italy) at 1:500 concentrations following the standard protocol. Sections were washed for 10 minutes in PBS plus 0.1% Triton® X-100. This step permeabilizes the tissue and it is required for optimal staining. After that, sections were washed two times for 5 minutes each in PBS. Diluted NeuroTrace has been used successfully. Approximately 200 *µ*L was applied to the slides, so that the sections were covered and incubated for 1 hour at RT. Subsequently the stain was removed and sections were washed for 10 min in PBS plus 0.1% Triton X–100. Brain sections were washed 2 times for 5 minutes each at RT in PBS. Hippocampi sections were mounted on gelatin-coated slides, coverslipped with GEL-MOUNT^TM^ and examined under an epi-illumination fluorescence microscope (Zeiss Axioskop 2) and a CLSM (Zeiss LSM 800). Neuronal area was measured using ImageJ software developed by Wayne Rasband (https://imagej.nih.gov/ij/docs/index.html). Briefly, we used a manual approach measuring the area of neurons, labeled with NeuroTrace, in the different area of hippocampus (CA1, CA3, DG) by drawing a region of interest (ROI) around them with one of the drawing tools (e.g., ellipse). From the Analyze Menu-Set measurements we selected “Area”, and finally we selected “Measure” from the Analyze Menu. The data obtained by image analysis were compared by means of two-way ANOVA including the group and the treatment as main factors.

#### 2.3.2. Evaluation of NIIs

Sections were stained with the antibody mouse anti-EM48 (MAB5374, Chemicon Temecula, CA) specific for the mutant huntingtin. Immunoreactivity was visualized using the goat anti-mouse Cy3-conjugated secondary antibody (Jackson Immunoresearch, West Grove, PA, USA). The images were acquired by CLSM (ZEISS LSM700) laser scanning confocal microscope. The number of NIIs in CA1, DG, and CA3 was calculated in each of three separate fields (one dorsolateral, one central, and one medial, each 1 mm in diameter) on each hemisphere in each of three rostrocaudally spaced sections from 9 mice from each experimental group.

#### 2.3.3. Immunofluorescence Analysis

Intensity of immunofluorescence was quantified using digital image analysis of the confocal images, acquired with a Confocal Laser Scanning Microscope Zeiss LSM700. For these experiments, all parameters during image acquisition were equal. All quantifications were performed using ImageJ software, developed by Wayne Rasband (http://imagej.nih.gov/ij/docs/index.html). Briefly, by a circle selection tool we selected all cells of interest. From the Analyze Menu-Set measurement we selected “Mean Grey Value”, “Area”, and “Min & Max Grey Value”. The “background” was subtracted choosing a region next to cells without fluorescence. Finally, we selected “Measure” from the Analyze Menu to obtain a mean value (expressed in arbitrary units/ Y-axis in the graphs).

#### 2.3.4. Western Blotting

Dissected hippocampi into the region CA1-CA3 and DG were homogenized with the RIPA lysis buffer containing a protease and phosphatase inhibitor cocktail (Sigma Aldrich, USA) and centrifuged at 13,000 g for 30 min. Equal proteins amounts were separated using sodium dodecyl sulfate-polyacrylamide gel electrophoresis, transferred to polyvinylidene fluoride membranes, and incubated with rabbit phospho-CREB (rabbit anti-Phospho-CREB, Upstate, NY) and mouse actin (1:10,000; Sigma Aldrich, St Louis, MO) antibodies, overnight at 4°C. After being washed with Tris-buffered saline (TBS)/Tween 20, membranes were incubated with HRP-labeled secondary antibody. Proteins signal was visualized using a Kodak Digital Science ID and quantified with ImageJ software 6.0.

#### 2.3.5. Immunoenzymatic Activity (ELISA)

Mice hippocampal concentration of mature BDNF into the different regions was measured performing the ELISA assay on (Immunological Sciences) supernatants of lysed tissues, following the protocol supplied by the manufacturer. Samples were diluted at the same proteins concentration in the appropriate buffer solution. The produced colorimetric reaction was measured at 450 nm using a microplate reader (Thermo Scientific Multiskan EX, Waltham, MA, USA). Each test was performed in triplicate and the BDNF concentrations are expressed as pg/mg of protein.

#### 2.3.6. Statistical Analysis

The data collected were analyzed to compare the effect of systemic BDNF in regard to hippocampal neurons area, NIIs number, BDNF protein expression, and CREB activation in each hippocampal region (CA1, DG, CA3) of differently treated groups. Statistical analysis was performed by one-way ANOVA for the NIIs number and two-way ANOVA between groups including genotype or treatment as principal factors, followed by Bonferroni posttest for multiple-comparison measures available on ImageJ software version 7.0.* P* values of less than 0.05 were considered to be statistically significant (see supplementary figures 2 and 3)

*Ethics Statement*. All studies were conducted in accordance with European Communities Council Directive of 24 November 1986 (86/609/ EEC) and approved by the Santa Lucia Foundation Animal Care and Use committee.

## 3. Results

### 3.1. Evaluation of Hippocampal Neurons Area

**Figure 1:**
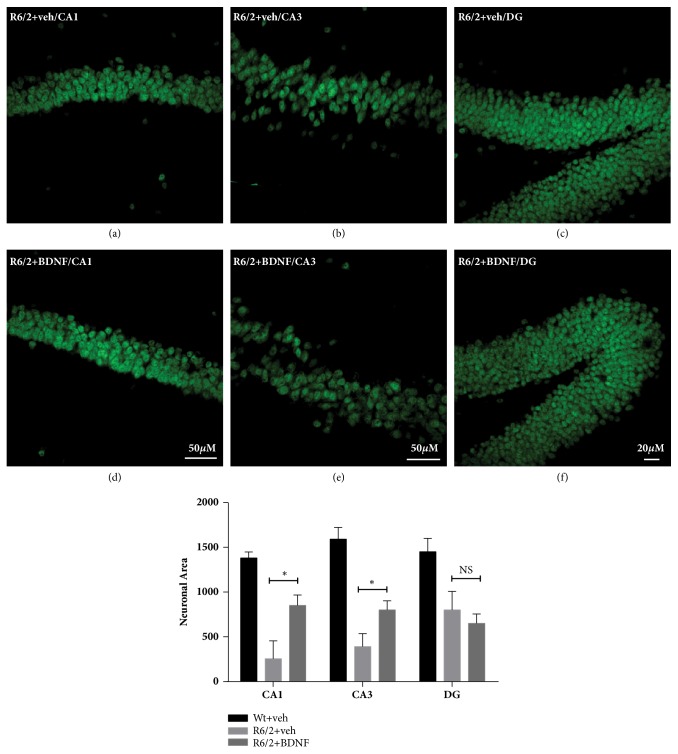
**Effects of BDNF treatment on the hippocampal neuronal area of wild type and R6/2 mice**. Representative confocal laser scanning microscopy images of single-label immunofluorescence for green fluorescent Nissl in CA1, CA3/2, and DG fields of hippocampus of vehicle treated R6/2 mice (a–c) and BDNF treated R6/2 mice (d–f). Two-way ANOVA, followed by Bonferroni test, revealed a decrease in hippocampal neuronal area in CA1, CA3/2, and DG of saline treated R6/2 compared to wild type vehicle mice  ^*∗*^p<0,05; histograms also show an increase in hippocampal neuronal area after treatment with BDNF in both CA1 and CA3/2  ^*∗*^ p<0,05. Scale bar 50 *µ*m (confocal scanning microscopy images were acquired using a 20x objective for DG and a 40x objective for CA1 and CA3/2).

The effect of BDNF treatment was examined in hippocampus sections stained with green fluorescent Nissl, a specific neuronal marker. Quantitative analysis using confocal microscopy confirmed that the average of neuronal area into the regions of CA1, CA3, and DG of the R6/2 mice hippocampus was indeed significantly smaller than in wild type mice [genotype effect CA1 F(1,28)=224.00 P<0.0001; CA3/2 F(1,28)=433.2 p<0.0001 and DG F(1,28)=191.0 p<0.0001]. Treatment with BDNF prevented this change in CA1 and CA3 [treatment effect CA1 F(1,28)=5.362 P=0.027; CA3/2 F(1.28)=5.962 P=0.0203]. Conversely, the treatment with BDNF was not effective in increasing the neuronal area in DG, where the size of neurons was comparable with that of vehicle- treated R6/2 mice [treatment effect F(1,28)=2.044 P=0.1625] (Figures 1(a)–1(c)).

### 3.2. Evaluation of NIIs

**Figure 2:**
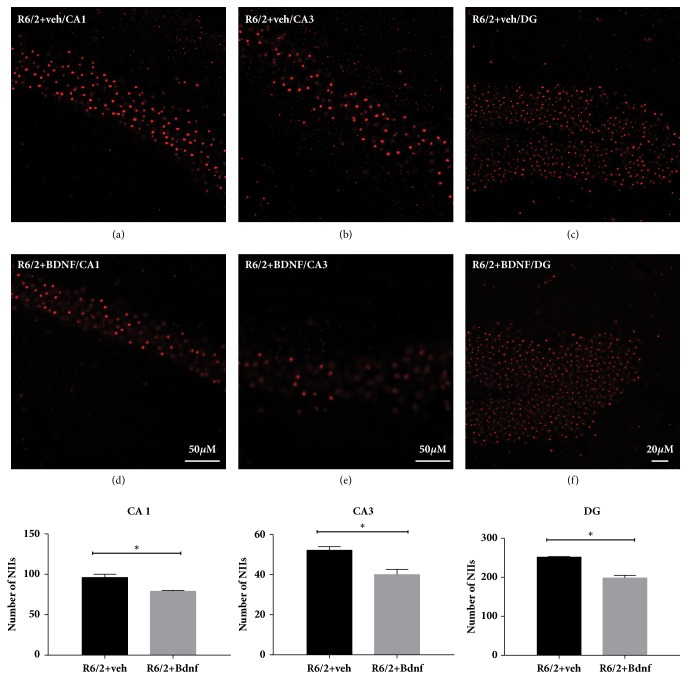
**Effects of BDNF treatment on the number of NIIs in the hippocampus of wild type and R6/2 mice**. Confocal laser scanning microscopy images of single-label immunofluorescence for NII marker (EM48). Single-label immunostaining was employed in the CA1, CA3/2 and DG fields of hippocampus of vehicle treated R6/2 mice and BNF-treated R6/2 mice (4 mg/day) at 13 weeks of age. Of note is the lower density of EM48-positive NIIs in the BDNF treated R6/2 mice. Quantification of NIIs in vehicle or BDNF treated R6/2 mice. There were no NIIs detected in hippocampus of wild type mice, so this group was not included in the statistical analysis. Scale bar 50 *µ*m (confocal scanning microscopy images were acquired using a 20x objective for DG and a 40x objective for CA1 and CA3.

The expression of exon 1 of mutant huntingtin in the R6/2 mice results in the formation of neuronal intranuclear inclusions (NIIs) detected with the antibody EM-48. EM-48 immunoreactivity was abundant in each hippocampal areas (CA1, CA3, and DG) from the vehicle treated R6/2 mice (Figures 2(b) and 2(e)). The average number of NIIs was significantly reduced in mice treated with BDNF (Figures 2(c) and 2(f)) (CA1 p<0,05; CA3 p<0,05; DG p<0,05). As expected, NIIs were not observed in wild type mice (supplementary figure 2).

## 4. Double Label Immunohistochemistry

### 4.1. Analysis of CREB in the Hippocampal Neurons

**Figure 3:**
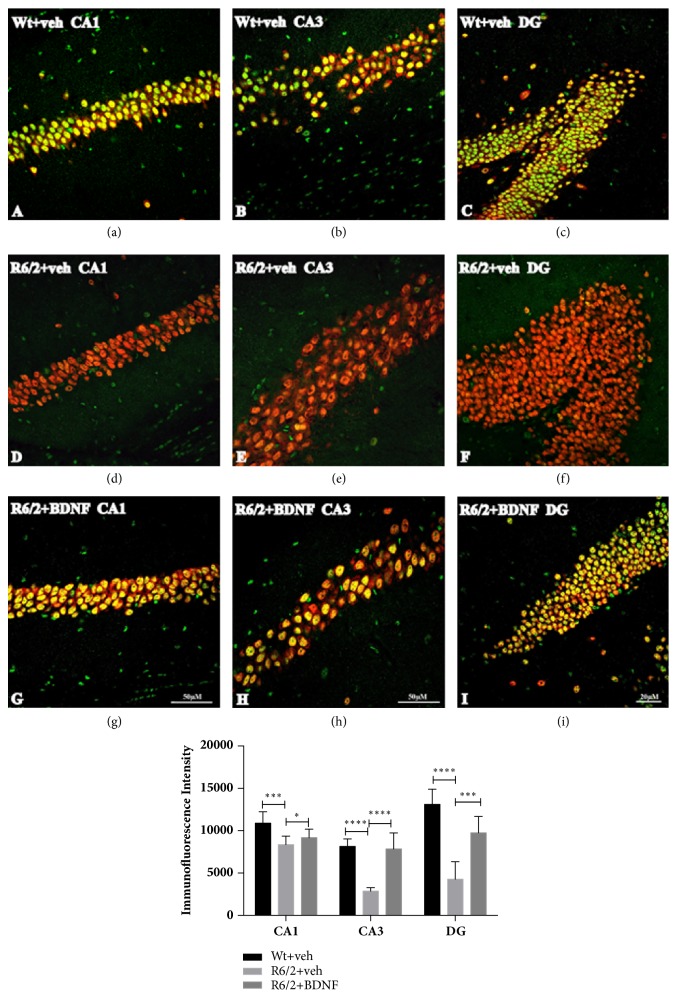
**Effects of BDNF treatment on pCREB protein expression in the hippocampus of wild type and R6/2 mice**. Representative confocal laser scanning microscopy images of double immunofluorescence for NeuN (visualized in red-Cy3 immunofluorescence) and pCREB (visualized in green-Cy2 immunofluorescence) in CA1, CA3/2, and DG of the hippocampus of wild type (a–c), R6/2 saline treated (d–f), and R6/2 BNDF treated (G-H) mice. Graph shows the quantification of the intensity of pCREB immunoreactivity associated with NeuN-labeled neuron in CA1, CA3/CA2 and DG hippocampal fields. Scale bar 50 *µ*m (confocal scanning microscopy images were acquired using a 20x objective for DG and a 40x objective for CA1 and CA3/2).

As shown in Figure 3 our immunohistochemical double labeling study revealed that the intensity of pCREB, expressed in arbitrary units, in the surviving hippocampal neurons was significantly decreased in the saline treated R6/2 in CA1 (Genotype effect F_(1,28)_=21,48 P=0,0003), CA3 (F_(1,28)_=43,70 p<0,0001), and DG (F_(1,28)_=85,95 p<0,0001) fields, compared to the wild type littermate (Figures 3(d)–3(f)). The levels of pCREB were significantly higher in systemic BDNF treated R6/2 compared to the saline R6/2 in CA3 (F_(1,28)_=36,07 p<0,0001) and DG (F_(1,28)_=16,67 p<0,0001) (Figures 3(g)–3(i)). Immunofluorescence data was confirmed by western blot assay, which revealed a statistically significant positive modulation of activated CREB in BDNF treated mice compared to vehicle treated R6/2 (Figures 5(a) and 5(b)).

### 4.2. Levels of BDNF in the Hippocampus

**Figure 4:**
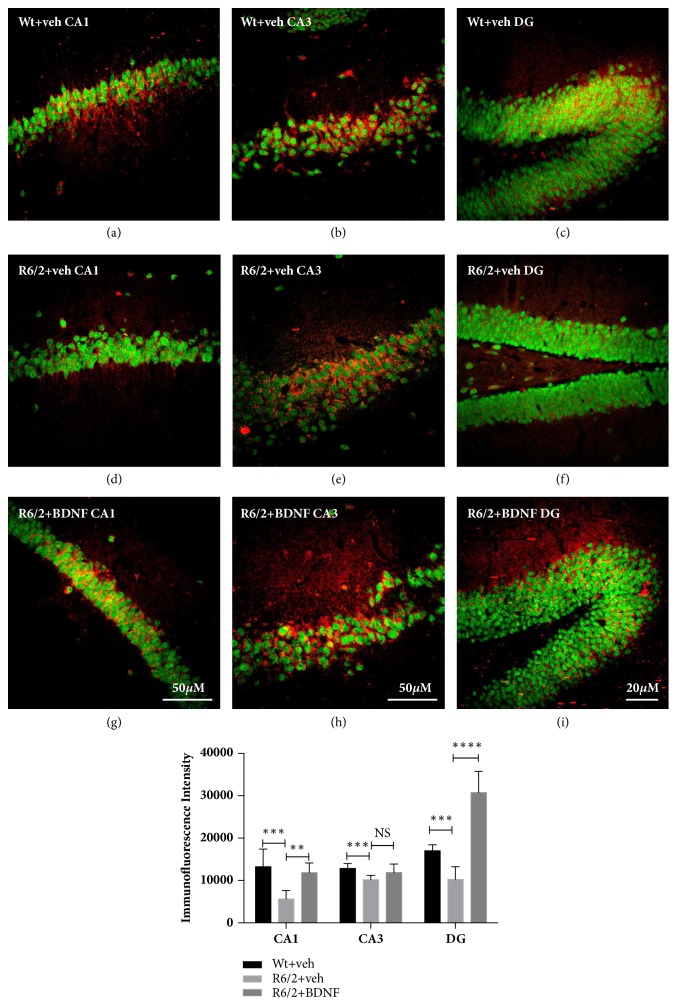
**Effects of BDNF treatment on BDNF protein expression in the hippocampus of wild type and R6/2 mice**. Representative confocal laser scanning microscopy images of double immunofluorescence for NeuN (visualized in green-Cy2 immunofluorescence) and BDNF (visualized in red-Cy3 immunofluorescence) in CA1 of the hippocampus of wild type (a–c), R6/2 saline treated (d–f) and R6/2 BNDF treated (g–i) mice. Graph shows the quantification of the intensity of BDNF immunoreactivity associated with NeuN-labeled neuron in CA1, CA3/CA2, and DG hippocampal fields. Scale bar 50 *µ*m (Confocal scanning microscopy images were acquired using a 20x objective for DG and a 40x objective for CA1 and CA3/2).

We aimed to investigate whether R6/2 mice saline treated shows an improvement in BDNF levels in CA1, CA3, and DG fields of hippocampus and whether the treatment with systemic BDNF affected BDNF expression in the hippocampus. As shown in Figure 4, the fields CA1 and DG of saline treated R6/2 mice were characterized by lower levels of BDNF compared to wild type mice (CA1 F_(1,28)_=16,68 P=0,0003; DG F_(1,28)_=11,73 p< 0,0017) (Figures 4(d)–4(f)).

**Figure 5:**
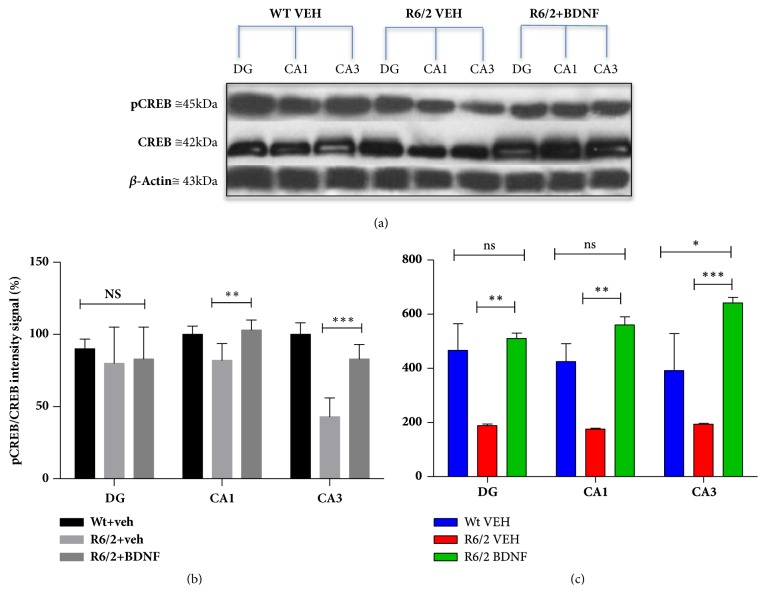
**Expression level of pCREB and BDNF in the hippocampus DG, CA1, and CA3 regions of R6/2 injected with BDNF.** Mice hippocampus was removed and the DG, CA1, and CA3 regions were isolated and lysate to perform western blot assay. Panels (a) and (b) show the decrease of pCREB expression levels, especially in the CA3 region and a statistically significant increment in BDNF treated R6/2,  ^*∗∗∗*^p<0,001. (c) Bonferroni posttest for repeated-measures comparison showed a significant increment of BDNF protein levels in treated-R6/2 DG and CA1 regions compared to vehicle treated mice,  ^*∗∗∗*^p<0,001.

BDNF treatment resulted in a significant increase in BDNF protein expression both in CA1 (F_(1,28)_=7,821 P=0,0003) and in DG (F_(1,28)_=98,81 p< 0,0001) of R6/2 compared to the saline R6/2 mice. Conversely, no significant changes in BDNF levels were observed in CA3 of R6/2 saline and BDNF treated compared to wild type mice (Figures 4(g)–4(i)). Levels of BDNF in the DG, CA1, and CA3 regions were investigated by ELISA assay. This confirmed our immunolabeling results, showing also a statistically significant modulation of BDNF in CA3. Because ELISA assay can detect the target protein in a concentration range 31.2-2000pg/ml, a significant modulation of BDNF was observed also in treated-R6/2 CA3 region compared to vehicle treated R6/2 (Figure 5(c)).

## 5. Discussion 

In this study, we observed that BDNF and CREB levels are decreased in the hippocampus of the R6/2 mouse model of HD. Moreover, systemic administration of BDNF was effective in ameliorating the neuropathology in the hippocampus of the R6/2 mouse model of HD. This neuroprotection was associated with an increased level of both phosphorylated CREB and BDNF. HD patients experience a severe cognitive decline, which often precedes the onset of motor symptoms. The deficits are typically attributed to disturbances in the corticostriatal system, but other structures involved in cognition, such as amygdala and the hippocampus, are affected in the early stages of the disease [5, 33]. Morphological changes in the hippocampus were reported in HD models [34, 35], as well as a deficit in hippocampal neurogenesis [36, 37]. A consistent cell loss in the hippocampus in HD was demonstrated preferentially in the CAl area [19], and the formation of NIIs was shown to progressively affect the hippocampus parallel to the striatum in HD models [21, 38].

In the present study, we demonstrated a reduction in the hippocampal neuronal area of vehicle treated R6/2 mice, thereby confirming previous observations [19]. Moreover, we showed that systemic BDNF treatment was able to rescue neurons, mostly in the CA1 and CA3 areas of the hippocampus. Similarly, we observed the formation of NIIs in the whole hippocampal area of vehicle treated R6/2 mice, as previously reported [21], and showed a reduction of NIIs in the BDNF treated R6/2 mice. Deficits in synaptic plasticity and memory were observed in several mouse models of HD [12, 27, 35, 39, 40]. Impaired learning has been described before motor symptoms and neuronal loss in HD mouse models, and they are associated with deficits in hippocampal long-term potentiation (LTP) [16], a form of synaptic plasticity which is considered a substrate for memory encoding, and by reduction in mossy fiber potentiation [41] and long-term depression [42]. Hippocampal function is severely altered in HD contributing to a complex degree of cognitive deficit as seen in HD patients [43]. Moreover, a cross-talk between the striatum and hippocampus should be taken into account, particularly in this pathological condition [44]. The deficit in plasticity is likely related to the evidence that mutant huntingtin decreases expression of BDNF and its TrkB receptor in neocortex and hippocampus in HD patients [23, 45] and mice [23, 46–49]. BDNF is a potent positive modulator of LTP when the potentiation effect is induced by naturalistic theta burst stimulation (TBS) [25]. BDNF modulates memory and cognitive alterations before the onset of motor symptoms in HD mice [39, 50]. Indeed, BDNF administration to hippocampal slices, or its up-regulation with ampakine, is able to rescue synaptic plasticity in the knock-in mouse model of HD [27, 40].

In the present study, we showed a reduction in BDNF protein expression in the R6/2 mice treated with vehicle compared to wild types. Systemic BDNF was able to increase BDNF protein levels significantly. To our knowledge, this is the first report of a decrease in hippocampal BDNF content in the R6/2 mouse model of HD. Previous studies had described that BDNF transcripts diminish during the course of the disease, particularly in the striatal area [51]. However, our study suggests an involvement of BDNF in the hippocampal dysfunction in the R6/2 mouse model. The transcription factor CREB is essential for activity-induced gene expression mediating memory formation [52]. Thus, CREB plays a major role in the hippocampus. Expression of CREB target genes and of the transcriptional coactivator CREB binding protein (CBP) was shown to be diminished in the hippocampus of HD mice [22]. In particular, a significant reduction in CREB target genes related to synaptic plasticity and memory such as c-fos, Arc, or Nr4a2 was observed. Thus, these changes were associated with long-term spatial and recognition memory deficits in the HD mouse model [22].

Here we report a decrease in phosphorylated CREB in the hippocampus of the R6/2 mouse. A selective deficit in long-term memory in mice carrying a mutation of CREB was demonstrated earlier [53]. Indeed, CREB signaling is a pathway critical for hippocampal-dependent synaptic plasticity and long-term memory [54]. Even though pCREB levels in the hippocampus were not found to be decreased in HD mice in a previous report [22], an increase in hippocampal pCREB was recorded after the administration of phosphodiesterase 10 (PDE10) inhibitors [55]. Systemic BDNF was able to increase pCREB in the hippocampus. The effect of increasing pCREB levels is consistent with the possibility that peripheral BDNF enters the brain and directly activates TrkB–ERK–CREB signaling [29, 30]. The neuroprotective role exerted by BDNF is thus confirmed. Our study thus confirms the involvement of pCREB in the hippocampal functions in HD. Moreover, the relation between hippocampal BDNF and pCREB is confirmed by our study, as CREB has a permissive role in BDNF gene expression [56, 57]. The beneficial effects of treatment with systemically administered recombinant BDNF is confirmed in the hippocampus [29, 30]. In particular, recombinant BDNF was able to ameliorate neuropathological features by significantly reduce both hippocampal atrophy and the number of NIIs [38] in all hippocampal subfields. Such observations suggest that the involvement of BDNF in HD is not only confined to the striatum and cortex. Mutant huntingtin alters BDNF function, which is responsible for the modulation of a major component of the dysfunction in learning and memory in mouse models of HD [39].

A behavioral analysis to assess cognitive effect of treatment with BDNF would be an important addition to corroborate our data. The R6/2 mouse model of HD is characterized by a rapidly evolving pathology, and motor impairment occurs fairly early. Motor impairment represents an obstacle for the evaluation of cognitive impairment, and it would make data hard to interpret. Our lab is currently working to implement the method of detection, so that later stages of the disease can be explored also from a cognitive point of view.

BDNF function is a key regulator factor for the cognitive deficits of HD. Thus, strategies aimed at increasing BDNF levels in the brain will be useful, not only for the survival of the striatum, which is the main target of the disease, but also to preserve hippocampal functions in HD.

## Data Availability

The data used to support the findings of this study are included within the article.

## Supplementary Materials

Supplementary Materials Fig. 1. Supplementary Figure 1 shows the Nissl fluorescent staining in the group of BDNF treated wild type mice. There are no statistically significant differences compared to saline treated wild type mice. Fig. 2. The supplementary figure shows the absence of neuronal intranuclear inclusion (NIIs) in the wild type mice treated with BDNF. Fig. 3. Immunohistochemistry experiments performed in BDNF treated wild type mice. Supplementary Figure 3 shows the immunostaining for pCREB and BDNF used for the statistical analysis.

## References

[1] Albin R. L., Tagle D. A. (1995). Genetics and molecular biology of Huntington's disease. *Trends in Neurosciences*.

[2] Albin R. L., Reiner A., Anderson K. D. (1992). Preferential loss of striato‐external pallidal projection neurons in presymptomatic Huntington's disease. *Annals of Neurology*.

[3] Wilson R. S., Como P. G., Garronl D. C., Klawans H. L., Barr A., Klawans D. (2008). Memory failure in Huntington's disease. *Journal of Clinical and Experimental Neuropsychology*.

[4] Huntington Disease Collaborative Research Group (1993). A novel gene containing a trinucleotide repeat that is expanded and unstable on Huntington’s disease chromosomes. *Cell*.

[5] Rosas H. D., Koroshetz W. J., Chen Y. I. (2003). Evidence for more widespread cerebral pathology in early HD: An MRI-based morphometric analysis. *Neurology*.

[6] Ferrer I., Kulisevsky G., Gonzalez A. (1994). Parvalbumin-immunoreactive neurons in the cerebral cortex and striatum in Huntington's disease. *Neurodegeneration*.

[7] Squire L. R., Ojemann J. G., Miezin F. M., Petersen S. E., Videen T. O., Raichle M. E. (1992). Activation of the hippocampus in normal humans: a functional anatomical study of memory. *Proceedings of the National Acadamy of Sciences of the United States of America*.

[8] Nestler E. J., Barrot M., DiLeone R. J., Eisch A. J., Gold S. J., Monteggia L. M. (2002). The neurobiology of depression. *Neuron*.

[9] Pittenger C., Duman R. S. (2008). Stress, depression, and neuroplasticity: a convergence of mechanisms. *Neuropsychopharmacology*.

[10] Ho A. K., Sahakian B. J., Brown R. G. (2003). Profile of cognitive progression in early Huntington's disease. *Neurology*.

[11] Lemiere J., Decruyenaere M., Evers-Kiebooms G., Vandenbussche E., Dom R. (2004). Cognitive changes in patients with Huntington's disease (HD) and asymptomatic carriers of the HD mutation. *Journal of Neurology*.

[12] Lione L. A., Carter R. J., Hunt M. J., Bates G. P., Morton A. J., Dunnett S. B. (1999). Selective discrimination learning impairments in mice expressing the human Huntington's disease mutation. *The Journal of Neuroscience : The Official Journal of the Society for Neuroscience*.

[13] Mazarakis N. K., Cybulska-Klosowicz A., Grote H. (2005). Deficits in Experience-Dependent Cortical Plasticity and Sensory-Discrimination Learning in Presymptomatic Huntington's Disease Mice. *The Journal of Neuroscience*.

[14] Van Raamsdonk J. M., Pearson J., Slow E. J., Hossain S. M., Leavitt B. R., Hayden M. R. (2005). Cognitive Dysfunction Precedes Neuropathology and Motor Abnormalities in the YAC128 Mouse Model of Huntington's Disease. *The Journal of Neuroscience*.

[15] Hodgson J. G., Agopyan N., Gutekunst C.-A. (1999). A YAC mouse model for Huntington's disease with full-length mutant huntingtin, cytoplasmic toxicity, and selective striatal neurodegeneration. *Neuron*.

[16] Murphy K. P., Carter R. J., Lione L. A. (2000). Abnormal synaptic plasticity and impaired spatial cognition in mice transgenic for exon 1 of the human Huntington's disease mutation. *The Journal of Neuroscience*.

[17] Usdin M. T., Shelbourne P. F., Myers R. M., Madison D. V. (1999). Impaired synaptic plasticity in mice carrying the Huntington's disease mutation. *Human Molecular Genetics*.

[18] Gil J. M., Mohapel P., Araújo I. M. (2005). Reduced hippocampal neurogenesis in R6/2 transgenic Huntington's disease mice. *Neurobiology of Disease*.

[19] Spargo E., Everall I. P., Lantos P. L. (1993). Neuronal loss in the hippocampus in Huntington's disease: A comparison with HIV infection. *Journal of Neurology, Neurosurgery & Psychiatry*.

[20] Gil J. M., Popovic N., Brundin P., Petersén A. (2004). Asialoerythropoietin is not effective in the R6/2 line of Huntington's disease mice. *BMC Neurosci*.

[21] Morton AJ., Lagan M. A., Skepper J. N., Dunnett S. B. (2000). Progressive formation of inclusions in the striatum and hippocampus of mice transgenic for the human Huntington's disease mutation. *Journal of Neurocytology*.

[22] Giralt A., Puigdellívol M., Carretón O. (2012). Long-term memory deficits in Huntington's disease are associated with reduced CBP histone acetylase activity. *Human Molecular Genetics*.

[23] Zuccato C., Ciammola A., Rigamonti D. (2001). Loss of huntingtin-mediated BDNF gene transcription in Huntington's disease. *Science*.

[24] Rex C. S., Lin C.-Y., Kramár E. A., Chen L. Y., Gall C. M., Lynch G. (2007). Brain-derived neurotrophic factor promotes long-term potentiation-related cytoskeletal changes in adult hippocampus. *The Journal of Neuroscience*.

[25] Bramham C. R., Messaoudi E. (2005). BDNF function in adult synaptic plasticity: the synaptic consolidation hypothesis. *Progress in Neurobiology*.

[26] Kramar E. A., Lin B., Lin C. Y., Arai A. C., Gall C. M., Lynch G. (2004). A Novel Mechanism for the Facilitation of Theta-Induced Long-Term Potentiation by Brain-Derived Neurotrophic Factor. *The Journal of Neuroscience*.

[27] Simmons D. A., Rex C. S., Palmer L. (2009). Up-regulating BDNF with an ampakine rescues synaptic plasticity and memory in Huntington's disease knockin mice. *Proceedings of the National Acadamy of Sciences of the United States of America*.

[28] Kuipers S. D., Trentani A., Tiron A., Mao X., Kuhl D., Bramham C. R. (2016). BDNF-induced LTP is associated with rapid Arc/Arg3.1-dependent enhancement in adult hippocampal neurogenesis. *Scientific Reports*.

[29] Giampà C., Montagna E., Dato C., Melone M. A. B., Bernardi G., Fusco F. R. (2016). Systemic delivery of recombinant brain derived neurotrophic factor (BDNF) in the R6/2 mouse model of Huntington's disease. *PLoS ONE*.

[30] Giampà C., Montagna E., Dato C., Melone M. A. B., Bernardi G., Fusco F. R. (2013). Systemic delivery of recombinant brain derived neurotrophic factor (bdnf) in the r6/2 mouse model of Huntington's disease. *PLoS ONE*.

[31] Giampà C., Middei S., Patassini S. (2009). Phosphodiesterase type IV inhibition prevents sequestration of CREB binding protein, protects striatal parvalbumin interneurons and rescues motor deficits in the R6/2 mouse model of Huntington’s disease. *European Journal of Neuroscience*.

[32] Giampà C., Laurenti D., Anzilotti S., Bernardi G., Menniti F. S., Fusco F. R. (2011). Inhibition of the Striatal Specific Phosphodiesterase PDE10A Ameliorates Striatal and Cortical Pathology in R6/2 Mouse Model of Huntington's Disease. *PLoS ONE*.

[33] Rosas A. K., Liu S., Hersch M. (2002). Regional and progressive thinning of the cortical ribbon in Huntington's disease. *Neurology*.

[34] Bayram-Weston Z., Jones L., Dunnett S. B., Brooks S. P. (2012). Light and electron microscopic characterization of the evolution of cellular pathology in YAC128 Huntington's disease transgenic mice. *Brain Research Bulletin*.

[35] Brooks S., Higgs G., Jones L., Stephen B. (2012). Longitudinal analysis of the behavioural phenotype in HdhQ92 Huntington's disease knock-in mice. *Brain Research Bulletin*.

[36] Ransome M. I., Renoir T., Hannan A. J. (2012). Hippocampal Neurogenesis, Cognitive Deficits and Affective Disorder in Huntington's Disease. *Neural Plasticity*.

[37] Simpson J. M., Gil-Mohapel J., Pouladi M. A. (2011). Altered adult hippocampal neurogenesis in the YAC128 transgenic mouse model of Huntington disease. *Neurobiology of Disease*.

[38] Wanderer J., Morton A. J. (2007). Differential morphology and composition of inclusions in the R6/2 mouse and PC12 cell models of Huntington’s disease. *Histochemistry and Cell Biology*.

[39] Giralt A., Rodrigo T., Martín E. D. (2009). Brain-derived neurotrophic factor modulates the severity of cognitive alterations induced by mutant huntingtin: Involvement of phospholipaseC*γ* activity and glutamate receptor expression. *Neuroscience*.

[40] Lynch G., Kramar E. A., Rex C. S. (2007). Brain-Derived Neurotrophic Factor Restores Synaptic Plasticity in a Knock-In Mouse Model of Huntington's Disease. *The Journal of Neuroscience*.

[41] Gibson J. R., Beierlein M., Connors B. W. (2005). Functional properties of electrical synapses between inhibitory interneurons of neocortical layer 4. *Journal of Neurophysiology*.

[42] Milnerwood A. J., Cummings D. M., Dallérac G. M. (2006). Early development of aberrant synaptic plasticity in a mouse model of Huntington's disease. *Human Molecular Genetics*.

[43] Ciamei A., Morton A. J. (2009). Progressive imbalance in the interaction between spatial and procedural memory systems in the R6/2 mouse model of Huntington’s disease. *Neurobiology of Learning and Memory*.

[44] Ghiglieri V., Sgobio C., Costa C., Picconi B., Calabresi P. (2011). Striatum–hippocampus balance: From physiological behavior to interneuronal pathology. *Progress in Neurobiology*.

[45] Ferrer I., Goutan E., Marín C., Rey M. J., Ribalta T. (2000). Brain-derived neurotrophic factor in Huntington disease. *Brain Research*.

[46] Zuccato C., Liber D., Ramos C. (2005). Progressive loss of BDNF in a mouse model of Huntington's disease and rescue by BDNF delivery. *Pharmacological Research*.

[47] Gines S., Seong I. S., Fossale E. (2003). Specific progressive cAMP reduction implicates energy deficit in presymptomatic Huntington's disease knock-in mice. *Human Molecular Genetics*.

[48] Ginés S., Bosch M., Marco S. (2006). Reduced expression of the TrkB receptor in Huntington's disease mouse models and in human brain. *European Journal of Neuroscience*.

[49] Spires T. L., Grote H. E., Varshney N. K. (2004). Environmental enrichment rescues protein deficits in a mouse model of Huntington’s disease, indicating a possible disease mechanism. *The Journal of Neuroscience*.

[50] Di Filippo M., Tozzi A., Picconi B., Ghiglieri V., Calabresi P. (2007). Plastic abnormalities in experimental Huntington's disease. *Current Opinion in Pharmacology*.

[51] Benn C. L., Fox H., Bates G. P. (2008). Optimisation of region-specific reference gene selection and relative gene expression analysis methods for pre-clinical trials of Huntington's disease. *Molecular Neurodegeneration*.

[52] Silva A. J., Kogan J. H., Frankland P. W., Kida S. (1998). CREB and memory. *Annual Review of Neuroscience*.

[53] Bourtchuladze R., Frenguelli B., Blendy J., Cioffi D., Schutz G., Silva A. J. (1994). Deficient long-term memory in mice with a targeted mutation of the cAMP-responsive element-binding protein. *Cell*.

[54] Chen G., Zou X., Watanabe H., van Deursen J. M., Shen J. (2010). CREB Binding Protein Is Required for Both Short-Term and Long-Term Memory Formation. *The Journal of Neuroscience*.

[55] Giralt A., Saavedra A., Carretón O. (2013). PDE10 inhibition increases GluA1 and CREB phosphorylation and improves spatial and recognition memories in a Huntington's disease mouse model. *Hippocampus*.

[56] Nair A., Vaidya V. A. (2006). Cyclic AMP response element binding protein and brain-derived neurotrophic factor: Molecules that modulate our mood?. *Journal of Biosciences*.

[57] Lee Y., Kim J., Jang S., Oh S. (2013). Administration of Phytoceramide Enhances Memory and Up-regulates the Expression of pCREB and BDNF in Hippocampus of Mice. *Biomolecules & Therapeutics*.

