# Supplementary figures and images for: Modulation of Phospho-CREB by Systemically Administered Recombinant BDNF in the Hippocampus of the R6/2 Mouse Model of Huntington's Disease
Source: Neurosci J. 2019 Feb 6;2019:8363274. doi: 10.1155/2019/8363274 (PMC6381568; doi:10.1155/2019/8363274)

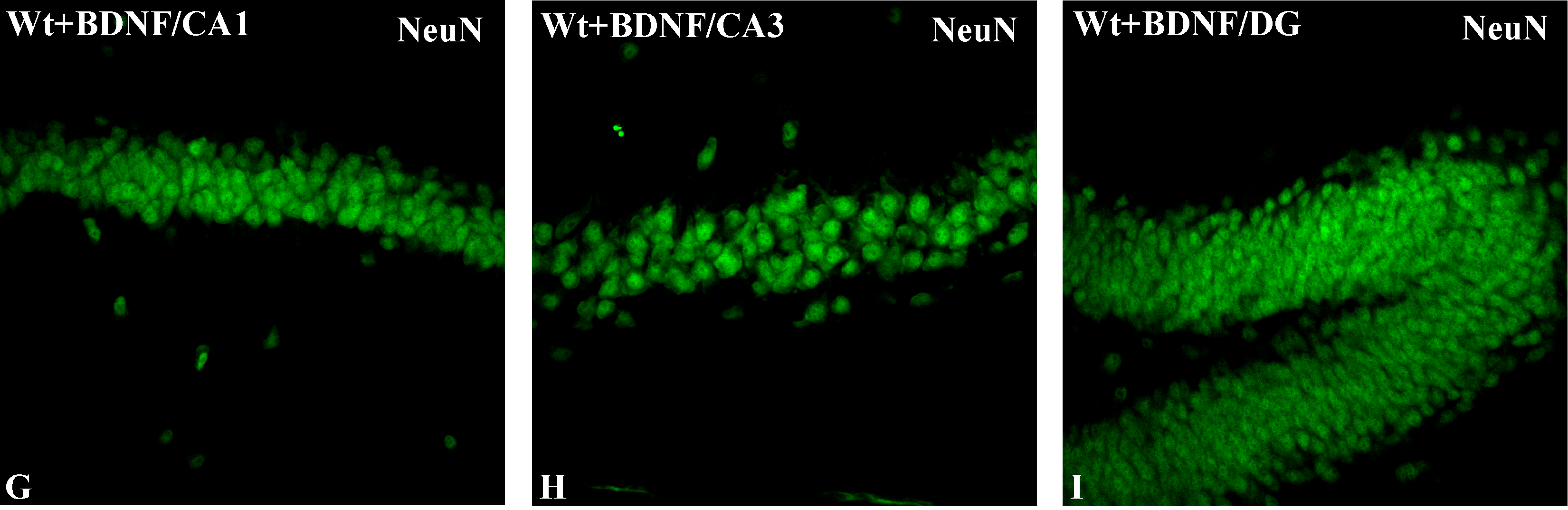

Supplement: Supplementary Materials — Fig. 1. Supplementary Figure 1 shows the Nissl fluorescent staining in the group of BDNF treated wild type mice. There are no statistically significant differences compared to saline treated wild type mice. Fig. 2. The supplementary figure shows the absence of neuronal intranuclear inclusion (NIIs) in the wild type mice treated with BDNF. Fig. 3. Immunohistochemistry experiments performed in BDNF treated wild type mice. Supplementary Figure 3 shows the immunostaining for pCREB and BDNF used for the statistical analysis. [file 8363274.f1.zip › Sup 1_NEUROSCIENCE_2632778.docx]

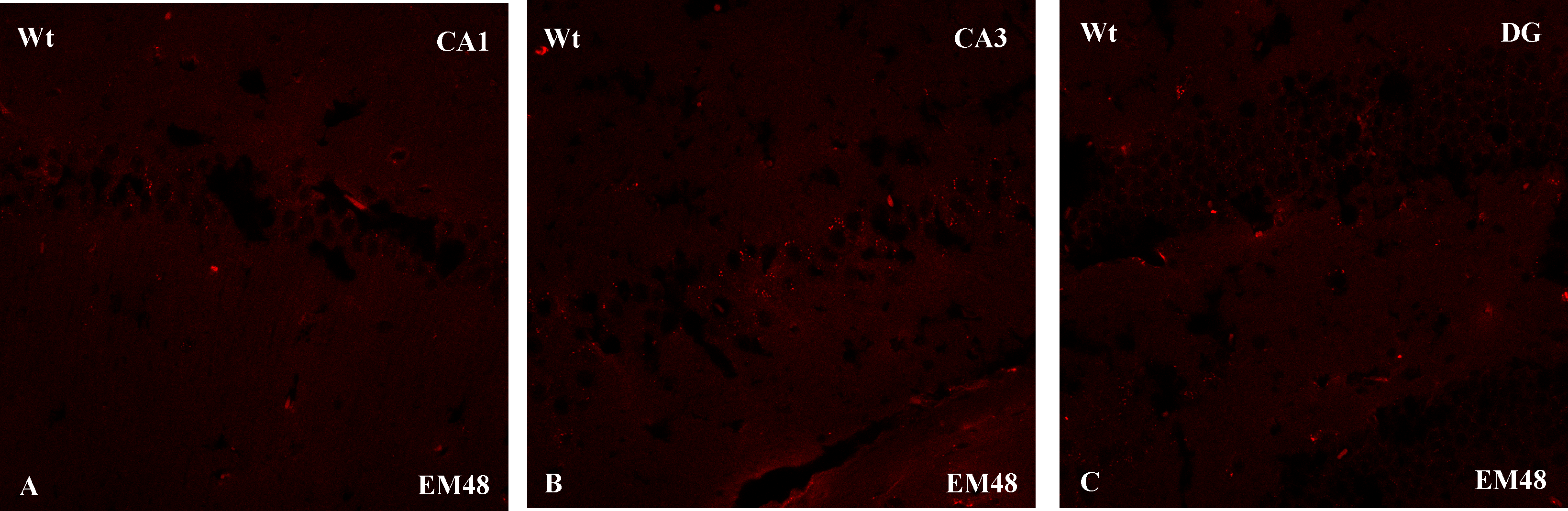

Supplement: Supplementary Materials — Fig. 1. Supplementary Figure 1 shows the Nissl fluorescent staining in the group of BDNF treated wild type mice. There are no statistically significant differences compared to saline treated wild type mice. Fig. 2. The supplementary figure shows the absence of neuronal intranuclear inclusion (NIIs) in the wild type mice treated with BDNF. Fig. 3. Immunohistochemistry experiments performed in BDNF treated wild type mice. Supplementary Figure 3 shows the immunostaining for pCREB and BDNF used for the statistical analysis. [file 8363274.f1.zip › Sup 2_NEUROSCIENCE_2632779.docx]

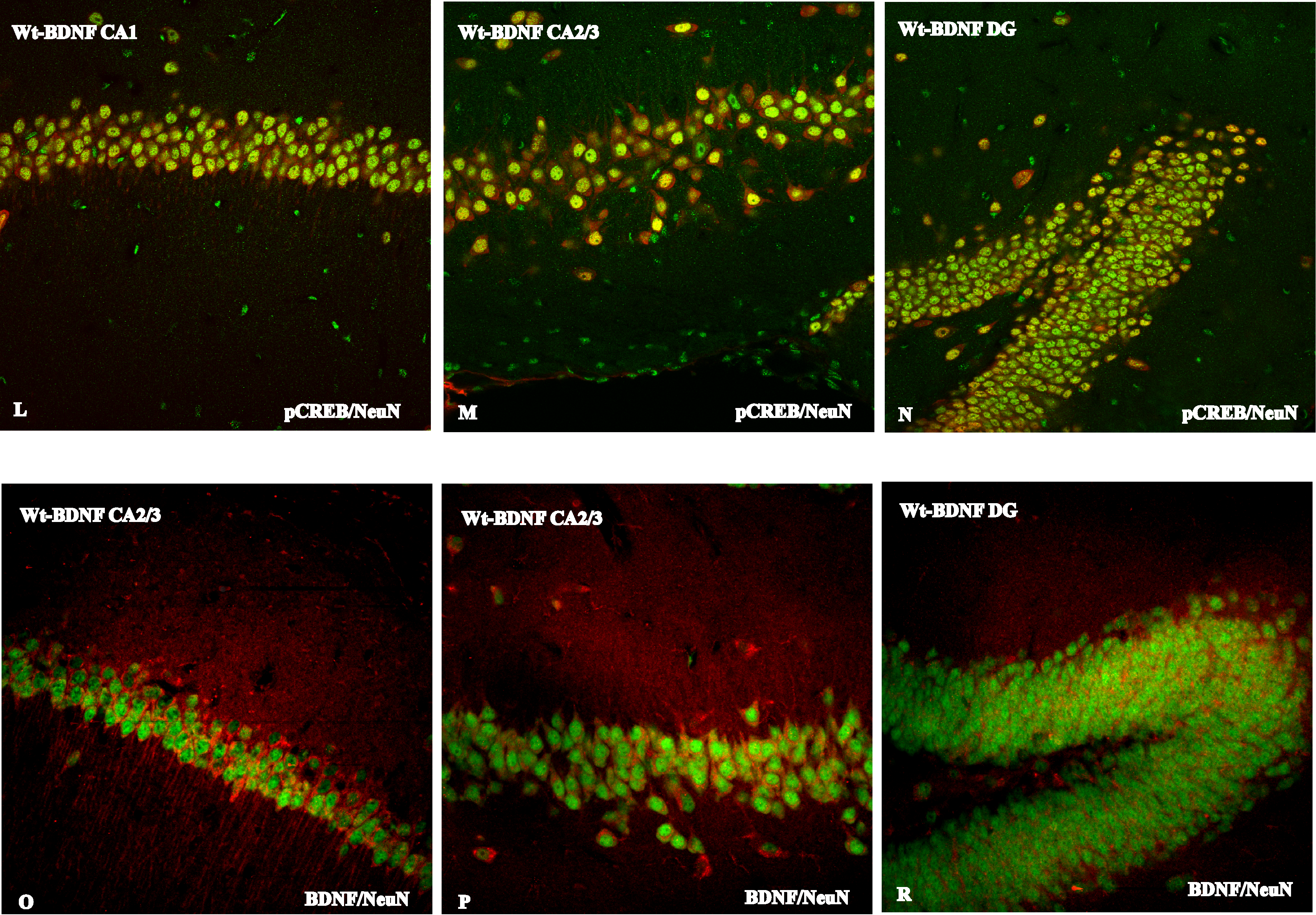

Supplement: Supplementary Materials — Fig. 1. Supplementary Figure 1 shows the Nissl fluorescent staining in the group of BDNF treated wild type mice. There are no statistically significant differences compared to saline treated wild type mice. Fig. 2. The supplementary figure shows the absence of neuronal intranuclear inclusion (NIIs) in the wild type mice treated with BDNF. Fig. 3. Immunohistochemistry experiments performed in BDNF treated wild type mice. Supplementary Figure 3 shows the immunostaining for pCREB and BDNF used for the statistical analysis. [file 8363274.f1.zip › Sup 3_NEUROSCIENCE_2632780.docx]
